# BicSPAM: flexible biclustering using sequential patterns

**DOI:** 10.1186/1471-2105-15-130

**Published:** 2014-05-06

**Authors:** Rui Henriques, Sara C Madeira

**Affiliations:** 1 Knowledge Discovery and BIOInformatics group (KDBIO), INESC-ID, and Computer Science and Engineering (CSE) Department, Instituto Superior Técnico, Universidade de Lisboa, Av. Rovisco Pais, 1, 1049-001 Lisboa, Portugal

## Abstract

**Background:**

Biclustering is a critical task for biomedical applications. Order-preserving biclusters, submatrices where the values of rows induce the same linear ordering across columns, capture local regularities with constant, shifting, scaling and sequential assumptions. Additionally, biclustering approaches relying on pattern mining output deliver exhaustive solutions with an arbitrary number and positioning of biclusters. However, existing order-preserving approaches suffer from robustness, scalability and/or flexibility issues. Additionally, they are not able to discover biclusters with symmetries and parameterizable levels of noise.

**Results:**

We propose new biclustering algorithms to perform flexible, exhaustive and noise-tolerant biclustering based on sequential patterns (BicSPAM). Strategies are proposed to allow for symmetries and to seize efficiency gains from item-indexable properties and/or from partitioning methods with conservative distance guarantees. Results show BicSPAM ability to capture symmetries, handle planted noise, and scale in terms of memory and time. BicSPAM also achieves the best match-scores for the recovery of hidden biclusters in synthetic datasets with varying noise distributions and levels of missing values. Finally, results on gene expression data lead to complete solutions, delivering new biclusters corresponding to putative modules with heightened biological relevance.

**Conclusions:**

BicSPAM provides an exhaustive way to discover flexible structures of order-preserving biclusters. To the best of our knowledge, BicSPAM is the first attempt to deal with order-preserving biclusters that allow for symmetries and that are robust to varying levels of noise.

## Background

Biclustering tasks over real-value matrices aim to discover sub-matrices (biclusters) where a subset of rows exhibit a correlated pattern over a subset of columns. However, existing approaches impose the selection of specific patterns of correlation, which often leads to incomplete solutions. A simple yet powerful direction to accommodate more flexible patterns – order-preserving patterns – was introduced by Ben-Dor et al. [1]. A bicluster is order-preserving if there is a permutation of its columns under which the sequence of values in every row is strictly increasing. These biclusters capture biclusters with shifting and scaling patterns of gene expression, and are, additionally, critical to detect other meaningful profiles as the progression of a disease or cellular response in distinct stages. Order-preserving biclustering can be applied to study gene expression (GE) data [2], genomic structural variations [3], biological networks [4], translational data [5,6], chemical data [7], nutritional data [8], among others [9,10]. Illustrating, subsets of genes that preserve the variation of expression levels for a subset of the conditions (either time-points, methods, stimuli, environmental contexts, tissues, organs or individuals) can disclose functional modules of interest.

Despite the relevance of the pioneer approach to find order-preserving biclusters (OPSM) [1] and of its extensions [11,12], this first class of *greedy approaches* suffers from two major drawbacks: *1)* delivers approximative solutions without optimality guarantees; and *2)* places restrictive constraints on the structure of the biclustering solutions (e.g. non-overlapping assumption). A second class of *exhaustive approaches*, *u*-Clustering (also known as OP-Clustering) [7,13], delivers solutions that overcome the flexibility issues of previous approaches. Still, their adoption presents three challenges: *1)* efficiency strongly deteriorates for matrices with more than 50 rows; *2)* noisy values lead to the partition of large biclusters in multiple smaller biclusters since they search for perfect orderings; and *3)* the use of non-condensed pattern representations leads to large biclustering solutions.

Additionally, the existing order-preserving approaches impose a monotonic ordering of values that does not allow for symmetries [1,7]. However, in biological domains, such as transcriptional activity analysis, regulatory and co-regulatory mechanisms are strongly correlated and, consequently, an increase in expression for some genes is sometimes accompanied by a decrease in expression for other genes.

This work introduces a new set of order-preserving biclustering approaches, referred as BicSPAM (Biclustering based on Sequential PAttern Mining), with principles to surpass the limitations of existing alternatives. BicSPAM promotes flexible and noise-tolerant searches, yet scalable, based on sequential patterns. BicSPAM contributions are three-fold: 

• [*Flexibility* ] Discovery of order-preserving biclusters with multiple levels of expressions and symmetries. Delivery of flexible structures of biclusters that allow for an arbitrary number and positioning of biclusters (to tackle the restrictive assumptions of greedy approaches);

• [*Robustness* ] Strategies for the discovery of biclusters with varying quality. Noise relaxations are made available to guarantee noise-tolerant solutions (to avoid the homogeneity restrictions imposed by existing exhaustive approaches), followed by filtering criteria to guarantee statistical significance of the discovered biclusters (to avoid the bias of greedy approaches);

• [*Efficiency* ] Scalable searches (to surpass efficiency limits of existing exhaustive approaches) based on new mining methods that seize efficiency gains from item-indexable properties of the biclustering task and from data partitioning principles.

Two additional contributions are provided: *1)* parameterizable selection of the degree of co-occurrences versus precedence relations observed in order-preserving biclusters; and *2)* strategies to handle missing values according to a parameterizable expectation of their appearance in biclustering solutions. Finally, BicSPAM integrates all the introduced principles into a coherent model that provides a consistent basis for the further development and extension of order-preserving biclustering approaches.

Experimental results on both synthetic and real datasets demonstrate the superior flexibility, robustness and effectiveness of BicSPAM. We also show the biological relevance of discovering order-preserving biclusters with symmetries.

The paper is organized as follows. The remainder of this section provides background on order-preserving biclustering and biclustering based on pattern mining. Methods section introduces BicSPAM. Results and discussion section validates the performance of BicSPAM against synthetic and real datasets. Finally, the contributions and implications of this work are synthesized.

### Order-preserving biclustering

#### 

**Definition 1.***Given a matrix, A *= (*X*,*Y*), *with a set of rows X *= {*x*_1_,..,*x*_*n*_}, *a set of columns Y *= {*y*_1_,..,*y*_*m*_}, *and elements *$a_{\text{ij}}\in R$*relating row i and column j*: 

• a ***bicluster*** *B *= (*I*,*J*) *is a r *× *s submatrix of A*, where *I *= (*i*_1_,..,*i*_*r*_) ⊂ *Xis a subset of rows and J*=(*j*_1_,..,*j*_*s*_) ⊂ *Yis a subset of columns;*

• the ***biclustering task*** *is to identify a set of biclusters*$ℬ=\{B_{1},\mathrm{..},B_{p}\}$*such that each bicluster B*_*k *_= (*I*_*k*_,*J*_*k*_) *satisfies specific criteria of homogeneity*, *where I*_*k*_ ⊂ *X*, *J*_*k*_ ⊂ *Yand*k∈N.

Biclustering approaches are driven by homogeneity criteria through the use of merit functions [2]. Merit functions either guarantee intra-bicluster homogeneity, the overall homogeneity of the output set of biclusters (inter-bicluster homogeneity), or both. Following the taxonomy proposed by Madeira and Oliveira [2], the existing biclustering approaches can be grouped acccording to their search paradigm, which determines how merit functions are applied^a^. The merit function is thus a simple way to define the type and quality of biclusters and to affect the structure of biclusters. The bicluster *type* defines the allowed pattern profiles and their orientation, the solution *structure* constrains the number, size and positioning of biclusters, and, finally, the *quality* determines the allowed noise within a particular or a set of biclusters. Biclusters can follow constant, additive, multiplicative or plaid pattern assumptions, either across rows or columns [1,2,8]. Multiple biclustering structures have been also proposed [2], with some approaches constraining them to exhaustive, exclusive or non-overlapping structures, and few others allowing a more flexible scheme with arbitrarily positioned overlapping biclusters.

Order-preserving biclusters were originally proposed for finding genes co-expressed within a temporal progression, such as co-expressions at particular stages of a disease or drug response [1]. However, its range of applications are equally attractive for matrices where time is absent. Illustrating, detecting relative changes in the expression of genes across conditions can be indicative of functional regulatory behavior and, additionally, surpasses the need to rely on the exact expression values that are usually noise-susceptible.

**Figure 1:**
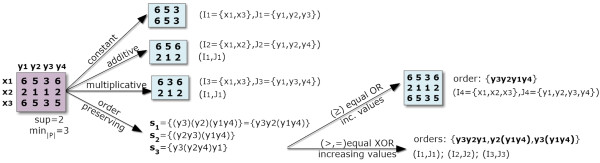
**Completeness and variants of order-preserving biclustering solutions.** Order-preserving biclusters have the power to capture flexible expression patterns – covering additive and multiplicative assumptions and additional profiles based on precedences and co-occurrences of expression values. They can be mined across rows or columns, and follow the or behavior (no differentiation between increasing and equality of ordered values) or the more specific xor behavior. A xor order-preserving bicluster requires that all of its rows share either an increasing or equality relation for the observed values of every pair of bicluster’s columns.

Order-preserving biclusters can emulate the majority of the previously introduced types of biclusters, leading to more inclusive solutions as illustrated in Figure 1. This offers a less restrictive setting to study larger functional modules associated with the discovered biclusters. Order-preserving biclusters can either allow monotonically increasing values (or behavior) or require strictly increasing values (xor behavior). In particular, when considering biclusters with monotonically increasing values, the permutation *π* = {*y*_3_,*y*_2_,*y*_4_,*y*_1_} in Figure 1 becomes supported by all rows {*x*_1_,*x*_2_,*x*_3_}. In fact, as illustrated in this figure, the flexibility of order-preserving biclusters is attractive as they cover constant, additive and multiplicative biclusters, which leads to more inclusive solutions.

#### 

**Definition 2. ***A bicluster following an order-preserving modelis* (*I*,*J*) *where J is a set of s columns respecting a π linear ordering, and I is the set of supporting rows where the s corresponding values are ordered according to the permutation π*.

There are two major types of approaches for order-preserving biclusters: greedy and exhaustive^b^. Exhaustive approaches aim to identify the largest submatrices where the set of rows are the maximum sets that support a linear order of values across the set of columns [7]. Contrasting, greedy approaches rely on a merit function to guide the composition of incrementally larger/smaller biclusters. The merit function used by the original greedy order-preserving approach, OPSM [1], is based on the upper-bound probability that a random data matrix contains a bicluster with more rows supporting it. Multiple extensions have been proposed over OPSM, including: the OPSM-RM method [11] to discover order-preserving biclusters from multiple matrices obtained from replicated experiments; the POPSM method [12] to model uncertain data with continuous distributions based on a probabilistic extent to which a row belongs to bicluster; and the MinOPSM method [14] that implements a variant of the order-preserving task.

The evaluation of order-preserving solutions does not significantly differ from the evaluation of traditional biclustering solutions. When considering the knowledge of hidden biclusters, relative non-intersecting area (RNIA) [15], match scores [3,16] and clustering metrics (e.g. entropy, recall and precision) have been adopted. RNIA [15] measures the overlap area between the hidden and found biclusters. *Clustering error* (CE) [17] extends this score to distinguish if several or exactly one of the found biclusters cover a hidden bicluster. Match scores (MS) [16] assess the similarity of solutions based on the Jaccard index. To turn MS sensitive to the number of biclusters in both sets, a consensus can be introduced by computing similarities between the Munkres pairs of biclusters [3].

In the absence of hidden biclusters, merit functions can be adopted as long as they are not biased towards the merit functions used within the approaches under comparison. Complementary, statistical evaluation has been proposed based on biclusters’ expected probability of occurrence [18,19] or based on their enrichment *p*-values against real datasets [20-22].

### Sequential pattern mining

Let an item be an element from an ordered set . An *itemsetp* is a set of non-repeated items, p⊆ℒ. A *sequences* is an ordered set of itemsets. A *sequence database* is a set of sequences *D*={*s*_1_,..,*s*_*n*_}.

Let a sequence *a*= < *a*_1_…*a*_*n*_ > be a *subsequence* of *b*= < *b*_1_…*b*_*m*_ > (*a*⊆*b*), if $\exists_{1\leq i_{1}<\mathrm{..}<i_{n}\leq m}:a_{1}\subseteq b_{i_{1}},\mathrm{..},a_{n}\subseteq b_{i_{n}}$. A sequence is *maximal* with respect to a set of sequences, if it is not contained in any of them. Illustrating, *s*_1_= < {*a*},{*be*} > = *a *(*be*) is contained in *s*_2_ = (*ad*) *c*(*bce*) and is maximal w.r.t. *D *= {*ae*,(*ab*) *e*}.

#### 

**Definition 3. ***The ***coverage***Φ*_*s*_*of a sequence s w.r.t. to a sequence database D is the set of all sequences in D for which s is subsequence: Φ*_*s*_={*s*^′^∈*D*∣*s*⊆*s*^′^}. *The ***support ***of a sequence s in D*, *denoted sup*_*s*_, *can either be absolute, being its coverage size* ∣ *Φ*_*s*_ ∣, *or a relative threshold given by* ∣*Φ*_*s*_∣/∣*D*∣.

To illustrate these concepts, consider the following sequence database *D*={*s*_1_=(*bc*)*a*(*abc*)*d*,*s*_2_=*cad*(*acd*),*s*_3_=*a*(*ac*)*c*}. For this database, we have ∣ℒ∣= ∣{*a*,*b*,*c*,*d*}∣ = 4,*Φ*_{*a*(*a* *c*)}_={*s*_1_,*s*_2_}, and *sup*_{*a*(*a* *c*)}_=2.

#### 

**Definition 4. ***Given a set of sequences D and some user-specified minimum support threshold θ*, *a sequence s*∈*D is frequent when contained in at least θ sequences. The ****sequential pattern mining*** *(SPM) problem consists of computing the set of frequent sequences,* {*s*∣*sup*_*s*_≥*θ*}.

The set of maximal frequent sequences for the illustrative sequence database, *D*= {(*bc*)*a*(*abc*)*d*,*cad* (*acd*),*a*(*ac*)*c*}, under the support threshold *θ*=3 is {*a*(*ac*),*cc*}. Existing SPM methods rely on (anti-) monotonic properties to efficiently find sequential patterns.

Consider two sequences *s* and *s*^′^, where *s*^′^⊆*s*, and a predicate *M*. *M* is *monotonic* when *M*(*s*)⇒*M*(*s*^′^) and *M* is *anti-monotonic* when ¬*M*(*s*^′^)⇒¬*M*(*s*). SPM approaches usually rely on these principles: the support of *s* is bounded from above by the support of *s*^′^ and if *s*^′^ is not frequent, then *s* is not frequent.

#### 

**Definition 5. ***Given a sequence database and a minimum support threshold θ*: 

• *a frequent sequence s is a sequence with*$|\;\Phi_{s}\;|\geq \theta$;

• *a closed frequent sequence is a frequent sequence that is not a subset of sequences with same support*$\left(\forall_{s^{\prime }⊃s}|s^{\prime }|<|s|\right)$;

• *a maximal frequent sequence is a frequent sequence with all supersets being infrequent,*$\forall_{s^{\prime }⊃s}|\;\Phi_{s^{\prime }}\;|<\theta$.

A frequent subsequence *s* is maximal if is frequent and all supersequences *s*^′^ (*s*⊆*s*^′^) are infrequent, while is closed if it is frequent and there exists no superset with the same support. Given the sequence database *D*= {(*bc*)*a*(*abc*)*d*,(*ac*),*cad*(*acd*),*a*(*ac*)*c*}, support *θ*=3 and constraint ∣ *s* ∣≥2, there are 2 maximal patterns ({*a*(*ac*),*cc*}), 3 closed patterns ({*a*(*ac*),(*ac*),*cc*}) and 5 simple patterns ({*a*(*ac*),*aa*,*ac*,(*ac*),*cc*}).

### Pattern-based biclustering

Pattern-based biclustering approaches rely on pattern mining methods and, therefore, use support, potentially combined with confidence-correlation metrics, as the merit means to produce biclusters. There are two major paradigms for pattern-based biclustering.

**Figure 2:**
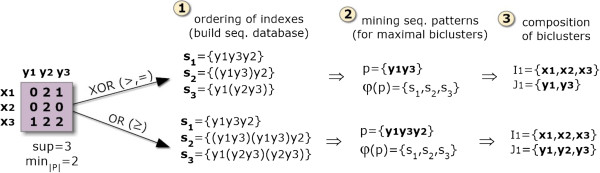
**Mining order-preserving biclusters in real or itemizespaced matrices.** To discover order-preserving biclusters the first step is to order the column indexes according to real or discretized values and map them to itemset sequences based on the observed ordering (precedences and co-occurrences). In particular, when targeting the or behavior, co-occurrences are propagated *n* times, being *n* the number of items co-occuring. Illustrating, *x*_2_= {*y*_1_=0, *y*_2_=2, *y*_3_= 0} is mapped as (*y*_1_*y*_3_)*y*_2_ sequence according to the xor behavior and as (*y*_1_*y*_3_)(*y*_1_*y*_3_)*y*_2_ under the or behavior. Second, a SPM method is applied over the set of sequences to extract the set of sequential patterns. Finally, biclusters are derived from the set of items and supporting transactions for each sequential pattern.

One option is to rely on sequential patterns [7,13] to produce order-preserving biclusters (Figure 2). These approaches follow a simple three-stage process. First, for each row, the column indexes are linearly ordered according to their expression values. Each row is, consequently, seen as a sequence of items that correspond to column indexes. Second, a SPM algorithm is applied over this set of sequences under a low support threshold for the discovery of frequent subsequences. Third, order-preserving biclusters are derived from the discovered sequential patterns – columns are derived from the subsequence’s items and rows from the set of sequences that support a frequent subsequence. This process can be easily adapted for an order-preserving assumption across rows by transposing both the input matrix and the generated biclusters.

Another option is to rely on frequent itemset mining [22-26]. Although these approaches only target biclusters with constant patterns, their analysis is critical as they provide key principles for flexible exhaustive searches. BiModule [27] allows for a parameterized multi-value itemization of the input matrix. DeBi [22] and Bellay’s et al. [28] place key post-processing principles to adjust biclusters in order to guarantee heightened statistical significance. GenMiner [23] includes external knowledge within the input matrix to derive biclusters from association rules.

## Methods

**Figure 3:**
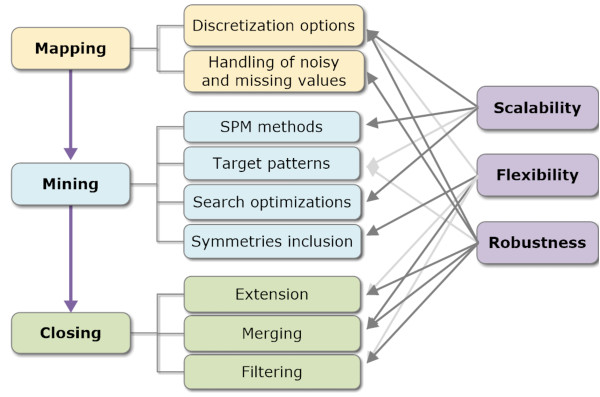
**BicSPAM methodology: major dimensions.** Principles to guarantee that BicSPAM approaches are scalable, flexible and robust to noise are addressed according three major steps. The *mapping* step defines the level and properties of the noise allowed through different discretization criteria and strategies to handle outliers and missing values. The core step, *mining*, defines structural performance aspects through the selection and parameterization of SPM methods. Finally, the *closing* step groups post-processing decisions to improve the quality and/or flexibility of biclustering solutions. BicSPAM methodology, thus, provides a roadmap to design and understand how the options associated to each step affect the performance of pattern-based approaches.

To tackle the scalability, flexibility and robustness issues of existing order-preserving approaches, we propose BicSPAM (Biclustering from Sequential PAttern Mining). BicSPAM defines key decision dimensions (Figure 3). Efficiency, flexibility and robustness of the target approaches are dependent on *mapping* (or pre-processing), *mining*, and *closing* (or post-processing) decisions. The *mapping* step consists on the itemization and re-ordering of the elements of the input matrix. The *mining* step, corresponds to the application of sequential pattern miners for the discovery of order-preserving biclusters. The *closing* step consists on the post-processing of the output patterns to affect the structure and quality of the target biclusters.

BicSPAM behavior section covers the fundamental options and structure of BicSPAM. The core contributions of BicSPAM are, then, conveyed in the following sections. Scalability, Flexibility and Quality sections provide critical principles and extensions to BicSPAM. Finally, Default and dynamic BicSPAM parameterizations section offers an integrated view of BicSPAM options and a method for their initialization based on data properties.

### BicSPAM behavior

Understandably, optimal and flexible solutions where the number and positioning of biclusters are not previously fixed require efficient search methods. SPM methods have been tuned during the last two decades according to scalability principles [29]. In this context, the composition of order-preserving biclusters from sequential patterns are a product of three steps (Figure 2). The columns of an input matrix are reordered according to their values, a SPM method is applied, and the output biclusters are mapped from the found frequent subsequences. Note that when two columns have equal values, they are seen as *co-occurrences*, while when their values differ they are treated as *precedences*. Consider the illustrative row *x*_2 _= {*y*_1 _= 0,*y*_2 _= 2,*y*_3 _= 0} in Figure 2, *y*_1_ and *y*_3_ co-occur, while *y*_1_ precedes *y*_3_. In this context, biclusters are derived from sequential patterns as follows:

#### 

**Definition 6. ***Given a matrix A and a minimum support threshold θ*, *a set of order-preserving biclusters* ∪_*k*_*B*_*k*_ where *B*_*k*_=(*I*_*k*_,*J*_*k*_) *can be derived from the set of frequent sequences* ∪_*k*_*s*^*k*^*by: 1) mapping*$\left(I_{k},J_{k})=(\Phi_{s^{k}},\{s_{i}^{k}|i=1\mathrm{..}|s^{k}|\}\right)$*to compose order-preserving biclusters on rows, or by 2) mapping*$\left(I_{k},J_{k})=(\{s_{i}^{k}|i=1\mathrm{..}|s^{k}|\},\Phi_{s^{k}}\right)$*from A*^*T*^*to compose order-preserving biclusters on columns.*

The support threshold defines the minimum number of rows in the bicluster. In the context of GE analysis, a low support is critical since significant co-expression patterns can occur for small groups of genes and/or conditions. Additionally, biclusters with a number of columns below a parameterizable threshold can be filtered by pruning subsequences with a number of items below that threshold. Finally, biclustering can either rely on the SPM methods as-is or target more dedicated searches by adapting the SPM support (merit function) and use it within the Apriori-based SPM framework. Existing support extensions include: Pandey et al. [24], Gowtham et al. [26], Huang et al. [30], and Steinbach et al. [31] measures. However, these metrics do not capture ordering relations and their definition needs to be (anti-)monotonic.

When the original numeric values are ordered without any form of discretization, the biclusters delivered by SPM-based methods are perfect biclusters, that is, they do not allow ordering mismatches. If discretization is applied with an ordinal alphabet, the number of co-occurrences per sequence increases. In this case, the output biclusters are not perfect but are naturally more robust to handle noise. The number of items in the considered alphabet can be used to control the level of noise-tolerance. However, discretization comes along with the drawback of potentially assigning two elements with similar values to different items. We refer to this drawback as the items-boundary problem.

**Figure 4:**
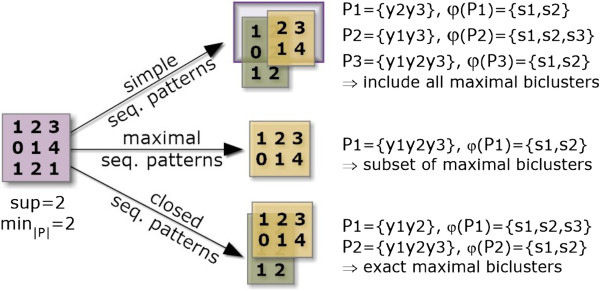
**Comparing biclustering solutions using simple, closed and maximal patterns.** Biclustering solutions derived from simple sequential pattern representations include all combinations of biclusters above a minimum support threshold (number of rows) and pattern length (number of columns). The adoption of maximal sequential patterns can lead to the loss of biclusters with a moderate number of columns but with a high number of rows since frequent sequences with fewer items but with a higher support are discard. Finally, approaches that use closed sequential patterns are the ones capable of returning all the maximal biclusters, the set of biclusters that are not totally included in another bicluster.

In particular, the chosen SPM method and target pattern representations affect the performance and output of the biclustering task. Contrasting with existing approaches, BicSPAM makes available alternatives for both variables aiming at an optimized behavior: 

• *SPM Methods:* Current *SPM methods* can be classified into three main categories: apriori-based, pattern-growth, and early-pruning [32]. Methods based on pattern-growth structures and early-pruning principle offer the best performance for the majority of biological data settings.

• Complementary to these search alternatives, both horizontal and vertical projections of the database are possible. Vertical projections for the SPM task are only competitive with the alternatives for very flattened matrices (*m*≫*n*). When targeting GE matrices, the methods that rely on vertical data formats should be only considered for the discovery of biclusters with order-preserving values on the rows (instead of columns). BicSPAM uses SPADE [33] (hybrid method) for vertical data settings (*m*≫*n*) and PrefixSpan [34] (pattern-growth method) for the remaining settings.

• *Pattern Representation:* The use of simple, closed or maximal patterns largely impact the properties of the biclustering solution, as illustrated in Figure 4. Efficiency gains can be seized when targeting condensed representations. *Maximal sequential patterns* lead to biclusters with the columns’ size maximized. However, since both vertical and smaller biclusters are loss, maximal-based biclusters lead to incomplete solutions. The alternative is to use *all sequential patterns* as in *μ*Cluster [7]. This solution leads to a high number of biclusters potentially redundant (if contained by another bicluster), which can degrade the performance of the mining and closing steps. Finally, *closed sequential patterns* allow for overlapping biclusters only if a reduction on the number of columns from a specific bicluster results in a higher number of rows. They are the target representation to obtain *maximal biclusters*, biclusters that cannot be extended without the need of either removing rows or columns. BicSPAM makes available CloSpan [35] and BIDEPlus [36] to mine condensed sequential patterns. Contrasting with existing approaches, closed sequential patterns (maximal biclusters) is the default option in BicSPAM.

**Figure 5:**
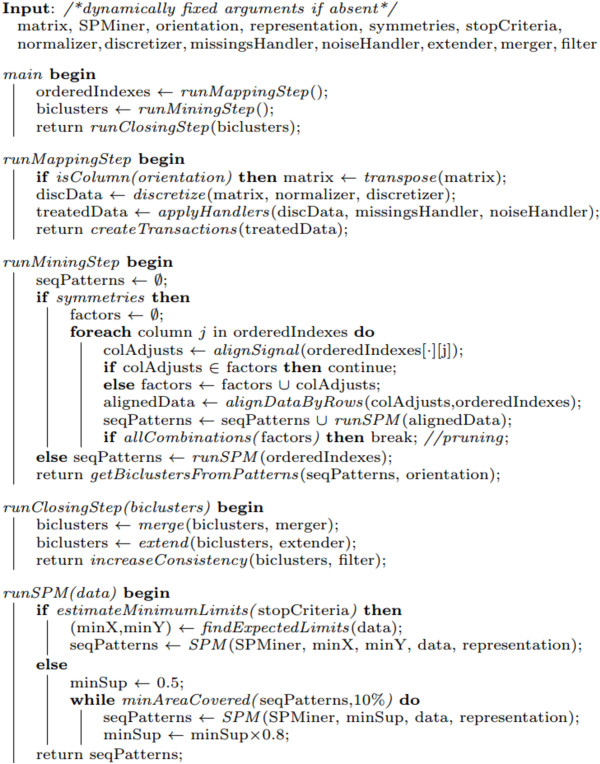
BicSPAM core steps.

The algorithmic basis of BicSPAM is provided in Figure 5 and described throughout the following sections. The computational complexity of BicSPAM is bounded by the SPM task and computation of similarities among biclusters for the closing options. Within the *mapping* step: outlier detection, normalization, discretization, noise correction procedures, distribution fitting tests and parameter estimations are linear on the size of the matrix, *Θ*(*nm*). The cost of the *mining step* depends on two factors: the complexity of the SPM method and on whether symmetries are allowed. The cost of the SPM task depends essentially on: the number and size of transactions (*γnm*, where *γ*≥1 captures the increase in size related with noise and missings handlers), the frequency distribution of items ({ℒ×Y}→N), the minimum support *θ*, the pattern representation, the chosen SPM method and on the presence of techniques to foster scabalibity (such as partitioning strategies). Let Θ(℘(γ,n,m,∣ℒ∣,θ)), or simply *Θ*(*℘*), be the complexity of the SPM task. The discovery of symmetries is pessimistically bounded by Θ(min(n2,m)×℘). Finally, the cost of the *closing* step, in accordance with the principles previously introduced by the authors [37], depends essentially on two factors: *1)* computing similarities among biclusters (required for merging and filtering biclusters), Θ(kk/2r¯s¯), where *k* is the number of biclusters and $\bar{r}\bar{s}$ their average size; and *2)* extending biclusters, $\Theta (k^{\prime }(\bar{r}m\;+\;n\bar{s}))$, where *k*^′^ is the number of biclusters after merging and filtering. The resulting complexity of BicSPAM is bounded by *Θ*(*hnm*+min(n2,m)℘+kk/2r¯s¯+k′(r¯m+ns¯)), which for datasets with a high number of patterns (*k*≫*k*^′^) is approximately Θ(min(n2,m)℘+kk/2r¯s¯).

### Scalability

Existing SPM methods are prepared to deal with sequences with an arbitrary repetition of items per sequence. However, order-preserved biclustering is derived from a more restricted form of sequences, item-indexable sequences, which do not allow item repetitions [13]. Additionally, a common input for the biclustering task is the minimum number of columns per bicluster, that is, the minimum number of items of the output sequential patterns. Although existing SPM methods can be applied in this context, they show inefficiencies to deliver large patterns due to the combinatorial explosion of sequential patterns under low support thresholds [13]. To avoid this, we propose two strategies to improve the scalability of BicSPAM. First, we extend IndexSpan algorithm [37] to discover sequential patterns with heightened efficiency from item-indexable sequences. Second, we propose the selection of specific mapping and closing options that foster the scalability of BicSPAM for large datasets.

#### Seizing item-indexable properties

IndexSpan [37], an extension on PrefixSpan [34], was previously proposed by the authors to seize efficiency gains from item-indexable databases (sequences without repeated items), while guarantee a narrow search space and efficient support counting. This method contrasts with *μ*Clusters method [7,13], which relies on a breadth search with high memory complexity *Θ*(*n*×*m*^2^) that does not scale for medium-to-large datasets (even in the presence of pruning techniques). IndexSpan considers the three following structural adaptations over the PrefixSpan algorithm. First, IndexSpan relies on an indexable compacted version of the original sequence database. Second, it uses faster and memory-efficient database projections, the most expensive step of PrefixSpan. Since the index of the items per sequence are known, IndexSpan projected database only maintains a list with the identifiers of the active sequences and of the prefix. To know if a sequence is still frequent when an item is added to a prefix, there is only the need to compare its index against the index of the previous item as well as their lexical order when the index is the same. Finally, the minimum number of items per sequential pattern, *δ*, is used to prune the search as early as possible. If the number of items of the current prefix plus the items of a postfix is less than *δ*, then the sequence identifier related with the postfix can be removed from the projected database since all the resulting patterns will have a number of items below the inputted threshold.

Two critical extensions over IndexSpan are implemented in BicSPAM. First, the discovered closed frequent sequences are represented within a compact tree structure, where the supporting transactions are annotated using principles proposed for full-pattern discovery [38]. Second, parameters from closing options are pushed to mining step. Illustrating, overlapping criteria for merging biclusters can be efficiently checked based on the properties of the tree, which significantly removes the complexity associated with computing similarities between all pairs of biclusters.

BicSPAM uses IndexSpan as the default SPM method due to its superior performance (against *μ*Clusters and traditional SPM methods) achieved by efficiency gains from fast database projections, minimalist data structures, and early pruning, merging and filter techniques.

#### Further efficiency options

The use of real-values or high number of items to define the orderings is an efficient option to find order-preserving biclusters as it guarantees a high number of precedences among column indexes (and low number of co-occurrences), leading to smaller sequential patterns. Contrasting, discretization with a low number of items is critical to guarantee more noise tolerant solution, but it degrades efficiency. This is due to the exponential increase of frequent sequential patterns either in number or size. To create a compromise between noise and efficiency, BicSPAM allows an arbitrary number of items and provides medium-to-high number of items as the default option (∣*Σ*∣≈*m*/5).

In this context, extending and merging of biclusters discovered using a high number of items can be applied to guarantee efficiency while preserving the quality of solutions. A second strategy is to increase the minimum support threshold (under a relaxed discretization more robust to noise) to promote an heightened SPM efficiency and the later application of filters to remove biclusters’ rows and columns in order to intensify their homogeneity. BicSPAM makes available extension, merging and filtering methods.

Finally, many of the principles proposed in the last decade to guarantee the scalability of SPM methods can be easily applied with IndexSpan. These principles include: data partitioning principles (inter- and intra-sequence), principles for the application of SPM methods in distributed settings, and the delivery of approximated sequential patterns (discovered under specific performance guarantees) [29,32].

### Flexibility

BicSPAM relies on flexible searches (no need to fix the number of biclusters apriori), delivers flexible structures of biclusters and allows for a flexible parameterization of its behavior (if a user opts not to use the dynamically learned parameters from data). In order to further guarantee the flexibility of the target BicSPAM approaches, we: *1)* extend the default order-preserving biclusters to allow for symmetric values, and *2)* define strategies to compose different structures of biclusters.

#### Order-preserving biclusters with symmetries

In GE analysis, allowing symmetries is required to combine regulatory and co-regulatory expression levels within a bicluster [24]. Two rows from a bicluster may have similar ordered levels of activity differing in sign. To our knowledge, this is the first attempt to combine symmetries with order-preserving models.

##### 

**Definition 7. ***A bicluster with symmetries is* (*I*,*J*) *with either symmetries on rows*${â}_{\text{ij}}=c_{i}\times a_{\text{ij}}$*or on columns*${â}_{\text{ij}}=c_{j}\times a_{\text{ij}}$, *where c*_*i*_∈{-1,1} *is the symmetry factor for each row of the bicluster and*$a_{\text{ij}}\in R$.

For the purpose of finding biclusters with symmetries, the normalization should satisfy the zero-mean criterion. Additionally, if the number of considered items for discretization is odd, there is one item being its own symmetric, which must be specially handled.

**Figure 6:**
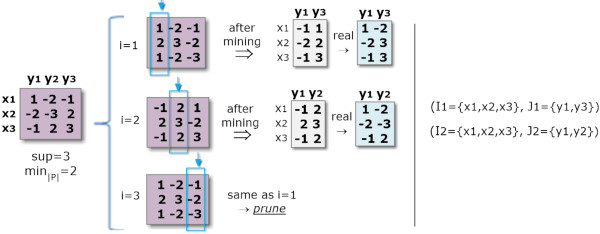
**Discovery of order-preserving biclusters with symmetries.** For each iteration the sign of expression of every row (or column) is coherently aligned in order to guarantee the consistency of signs for a target column (or row). Illustrating, *x*_2_ and *x*_3_ vectors were multiplied by -1 factor to guarantee the consistency of signs for *y*_1_ column. The target biclustering approach is then applied over this revised matrix. Iterations end when all the sign combinations have been covered.

The proposed method to find order-preserving biclusters allowing for symmetries is based on iterative sign corrections. If the goal is to find order-preserving coherency on the rows, then there is one iteration for each column *y*_*j*_. Within each iteration *j*, each row *x*_*i*_ is either multiplied by a 1 or -1 factor in order to guarantee that the observed values for the *y*_*j*_ column have the same sign. After the correction of the sign for each row, mining and closing steps are applied, the discovered biclusters are added to the solution set, and the method proceeds with the next iteration (column *y*_*j*+1_). Figure 6 illustrates this strategy.

Although the alignment of signs can be applied for every column *y*_*j*_, additional efficiency can be achieved by stopping the search when all the sign combinations have been achieved. Nevertheless, the worst case requires the application of a pattern miner *m* times. Note that filtering is a critical post-processing step to remove potential duplicates resulting from the repetition of coincident alignments.

#### Flexible biclustering structures

Pattern-based biclustering approaches produce highly flexible structures of biclusters. A pattern-based structure of biclusters allows overlaps and is non-exhaustive and non-exclusive. Additionally, the application of closing options over these structures allow the composition of structures with different properties, such as structures without overlapping areas. Shaping biclustering structures has been poorly addressed in literature, and rather seen as the byproduct of a target biclustering method [2].

Extension and merging of biclusters can be adopted to produce exhaustive structures (either overall, across rows or across columns). Filtering of exhaustive structures can be used to compose exclusive structures (either overall, across rows or across columns). BicSPAM makes available these closing techniques, that can be used to shape solutions with arbitrarily positioned biclusters. The composition of alternative structures in BicSPAM can be performed with sharp usability since there is no need to change the core mapping and mining steps.

### Quality

BicSPAM approaches are extended in this section regarding their robustness. Multiple mapping and closing options are proposed to handle missing values and deal with varying levels of noise.

#### Handling varying levels of noise

**Figure 7:**
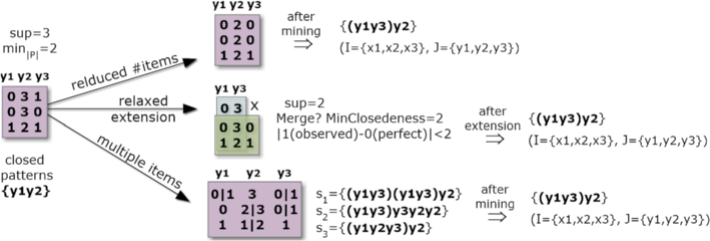
**Strategies to deal with varying noise relaxations.** Three strategies are illustrated. First, a relaxation is achieved by reducing the number of items of the alphabet from 4 to 3 items. Second, a lower support (*sup*=2) is combined with closing options to compose the final biclusters. In this example, this lower support leads to ({*x*_1_,*x*_2_,*x*_3_},{*y*_1_,*y*_2_}) and ({*x*_2_,*x*_3_},{*y*_1_,*y*_2_,*y*_3_}) biclusters, which can be extended or merge as a single larger bicluster ({*x*_1_,*x*_2_,*x*_3_},{*y*_1_,*y*_2_,*y*_3_}). Third, multiple items can be assigned per element using the distance between its value and the centroid of items. Illustrating, let *a*_1,1_=0.5, the centroid of items 0 and 1 be respectively 0.2 and 1.1, and the distance threshold be 0.7, then *a*_1,1_ is assigned to both 0 and 1 items (1.1 -0.7<*a*_1,1_<0.2+0.7).

A key direction to order-preserving biclustering is to consider multiple levels of noise by following one of the three strategies illustrated in Figure 7. *First* strategy, reduced number of items, hierarchically joins contiguous values to mine biclusters over matrices with varying levels of discretization. *Second* strategy, relaxed-to-restricted extensions under a lower support, considers varying levels of noise only after the mining. For instance, the merging of order-preserving biclusters can follow a statistical test sensitive to the closeness of original or discretized values. *Third* strategy, multiple items, associates one or more items to each element based on a parameterized threshold. This is critical to avoid the item-boundary problem (having a value near a frontier of discretization between two items). Different criteria can be defined to assign a varying number of items per element *a*_*i* *j*_. Each element can have two-to-three items based on the distance to their centroids. As a result, this method leads to sequences with multiple sizes, where column indexes can appear repeatedly within one sequence. If repetitions are observed for a specific sequential pattern, they are ignored during the definition of biclusters from that pattern.

#### Handling missing values

**Figure 8:**
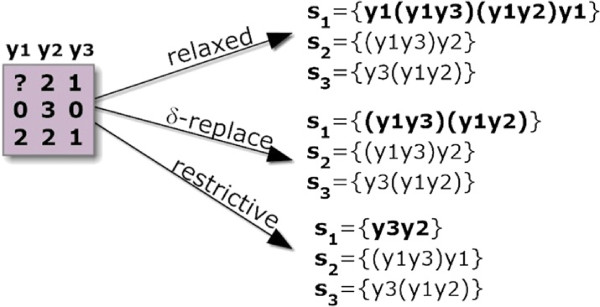
**Comparison of strategies to handle missing values.** In *relaxed* settings to handle missing values, the column index where a missing occurs is included as a co-occurrence in all positions of the respective sequence. Illustrating, *s*_1 _= *y*_3_*y*_2_ is mapped as *y*_1_(*y*_1_*y*_3_)(*y*_1_*y*_2_)*y*_1_. *δ*-replace setting is a more conservative alternative as it considers the inclusion of its index only in positions differing less that *δ* from its value-estimation. If *a*_1,1_ is estimated to have value 1.5 and *δ*=0.5, then *a*_1,1_∈{1,2} and (since *y*_3 _= 1 and *y*_2 _= 2) *s*_1_ is defined as (*y*_1_*y*_3_)(*y*_1_*y*_2_). Finally, the more conservative method, *restrictive* setting, removes missing items from the corresponding transactions.

Input matrices can have missing values, a common case with GE matrices. One missing value not properly treated may result in the loss of rows and columns across one or more biclusters, which can contain critical information. Three different strategies can be applied to treat missing values: *i)* removal, *ii)* replacement, and *iii)* handling as a special value. The simplest method is to remove the containing row or column (usually the dimension with smaller size). In order not to loose other information critical to compose biclusters, a special item can be used to replace missing values, that is removed during the ordering of columns. In this way, each row can have a varying number of columns. Alternatively, many hole-replacing methods have been proposed [39-41], which alleviate the referred problem, but also introduce additional noise that can significantly decrease the homogeneity of the output biclusters. For this reason, we propose the use of an additional item that is specially handled according to a level of relaxation defined by the user, as illustrated in Figure 8. The lowest constrained setting (*relaxed*) replaces the missing element by all items. This is a radical alternative to guarantee that potentially relevant biclusters are not lost due to the presence of missing values. The medium constrained setting (*δ*-replace) considers multiple items around its value-estimation. The highest constrained setting (*restrictive*) removes missing items.

#### Robustness recurring to mapping options

BicSPAM allows for the application of normalization and discretization methods on the rows, columns or overall matrix. Each context leads to different biclusters and is, respectively, suited to find patterns on bicluster’s columns, rows or on both dimensions. Normalization options are used to scale and enhance differences on the values, which are critical when mining order-preserving regularities. Marcilio et al. [42] compare three normalization procedures for GE data: z-score, scaling and rank-based procedures. Additional normalization criteria have been reported [43,44]. BicSPAM requires zero-mean thus allowing for symmetries and providing a simple setting for the application of multiple probabilistic distributions. When assuming the presence of missing and outlier elements, a masking bitmap can be adopted for their exclusion [27].

**Figure 9:**
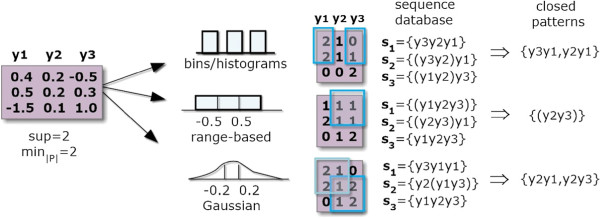
**Comparing BicSPAM discretization options.** The use fixed ranges is the simplest discretization option, but commonly leads to an accentuated weak distribution of items and is prone to the items-boundary problem. Percentage-based method tackle this observation using a depth partitioning of items that leads to intervals containing approximately the same number of items. Finally, alternative distributions (as the illustrated Gaussian) can be adopted to combine the properties of the previous solutions. Although Gaussian distributions are typically selected, Poisson distributions with a considerable number of occurrences (*λ*≥3) are dynamically selected for datasets without symmetric distribution of values around the median value. As illustrated, these methods can lead to biclustering solutions with heightened differences.

The applied discretization determines the weight of co-occurrences and precedences per sequence and, consequently, it has a strong influence on the output biclustering solutions. Although discretization implies loss of real distances among columns, it alleviates the noise dilemma [45,46]. BicSPAM allows for this control using two parameters: the number of items and the discretization method. Increasing the number of items decreases the number of co-occurrences and, therefore, reduces the noise-tolerance for elements with closer values but no significant ordering constraint. As a result of the stricter noise-tolerance, the output solutions tend to be composed by a larger number of biclusters with smaller areas. Additionally, BicSPAM makes available range-based, equal-depth partitioning and Gaussian cut-off points methods for discretization (default option), illustrated in Figure 9.

#### Robustness recurring to closing options

• *Merging Options*[28,47]. Merging methods allow for the delivery of noise-tolerant biclusters, thus recovering lost rows and columns due to the items-boundary problem or with missing/noisy values. An effective criterion to guide the merging is the overlapping area (as a percentage of the smaller bicluster), the default option in BicSPAM, or alternatively the resulting homogeneity of the bicluster after the merging.

• *Filtering Options*[22,27]. BicSPAM allows filtering at two levels: *1)* at the bicluster level and *2)* at the row-column level. For the first type of filtering, removal of biclusters that are duplicated or contained in larger biclusters, BicSPAM follows BiModule [27] heuristics to efficiently perform this type of filtering. The second type of filtering can be adopted to exclude rows or columns from a particular bicluster in order to intensify its homogeneity. This is usually the case when a low number of items is considered, leading to highly noise-tolerant biclusters. For this purpose, BicSPAM offers three strategies: *1)* use of statistical tests on each row and column, *2)* rely on existing greedy-iterative approaches and maximize their merit functions, and *3)* discover sequential patterns under more restrictive conditions (as higher support and confidence thresholds).

• *Extension Options*[22,28]. Similarly to filtering options at the row-column level, BicSPAM imple- ments three non-exclusive strategies to extend biclusters in ways that the resulting solution still satisfies some pre-defined homogeneity. First strategy relies on the use of greedy methods and on their merit functions for further extensions. Second strategy consists on the use of statistical tests to include rows or columns over each bicluster. Finally, BicSPAM provides a third novel strategy based on the merging of sequential patterns discovered under more relaxed support thresholds.

### Default and dynamic BicSPAM parameterizations

BicSPAM parameters with impact on the solution quality and efficiency are: 

• Mapping step parameters, including: the number of items (allowed noise), the normalization and discretization methods, and the (optional) methods to handle missing and noisy values;

• Mining step parameters, including: the inputted minimum number of rows and columns; the SPM method and its scalability extensions; and the chosen pattern representations;

• Closing step parameters, including the criteria to merge, filter and extend biclusters.

BicSPAM makes available default parameterizations (data-independent setting) and dynamic parameterizations (data-dependent setting). Default parameterizations include: zero-mean row-oriented normalization, overall Gaussian discretization with $\frac{m}{4}$ items (for an adequate trade-off of precedences vs. co-occurrences), and the use of row-based IndexSpan with closed sequential patterns, noise relaxation (allocation of 2 items for values in range *c*∈*a*,*b* with $\frac{\text{min}(b-c,c-a)}{b-a}<10\%$), removal of missing values and merging procedure with 80% overlapping. For the default setting, BicSPAM iteratively decreases the support threshold 10% (starting with *θ*=50%) until the output solution discovers 50 non-similar biclusters or a coverage of 10% of the elements in the input matrix.

The dynamic parameterizations adopt identical mining options but differ in the following aspects. Different distributions underlying the input matrix are tested to select the normalization and discretization procedure. If the range of values per row/column cannot be clustered with low error (within-cluster sum of squares), extension and filtering (at the column/row level) options are adopted to foster the robustness of BicSPAM. Moderate and relaxed missing handlers are selected if the input matrix has, respectively, over 2% and 5% of missing elements. Vertical searches using SPADE SPM method [33] are selected when *m* > 10*n*. Data partitioning principles to foster scalability are made available when the following condition is not satisfied: (*n*<20000∧*m*<100)∨(*n*<4000∧*m*<200).

These parameterizations provide a robust and user-friendly environment to use BicSPAM, while expert users can still further explore alternative behavior to obtain exploratory solutions with varying quality.

## Results and discussion

This section synthesizes the results from experimentally assessing the performance of BicSPAM. Results show that the proposed approaches are computationally efficient, flexible and robust to varying input settings. The methods were implemented in Java (JVM version 1.6.0-24). The experiments were performed using an Intel Core i5 2.30 GHz with 6 GB of RAM.

The experimental results are collected and analyzed in three steps. First, the impact of alternative BicSPAM parameterizations is analyzed in-depth for synthetic datasets with varying size, noise and sparsity. Second, the performance of BicSPAM is assessed against existing alternatives. Finally, the significance of BicSPAM results in biological contexts is assessed.

### Results in synthetic data

To study the performance of BicSPAM, two sets of datasets were generated. First, a set of synthetic matrices was generated using the experimental settings described in Table 1. We varied the size of these matrices (maintaining the proportion between rows and columns commonly observed in gene expression data) up to 2.000 rows and 100 columns. The number and shape of the planted biclusters were also varied. The number of rows and columns for each bicluster follows an Uniform distribution over the ranges presented in Table 1. The Uniform selection allows for repetitive choices, thus creating overlaps among biclusters, which can harden the recovery of the planted biclusters. Finally, a noise factor (up to ±10% of the domain range) was applied to each bicluster. For each of these settings we instantiated 20 matrices: 10 matrices with background values from the continuous Uniform distribution *U*(-1,1) and 10 matrices with background values generated according to the Gaussian distribution *N*(*μ*=0,*σ*=1). The presented results are an average across these 20 matrices.

**Table 1:** Properties of the generated dataset settings

Matrix size (*♯rows*×*♯*cols)	100 × 30	500 × 50	1000 × 75	2000 × 100
Number of hidden biclusters	2	3	5	8
Number of rows	[10,20]	[40,70]	[100,150]	[200,300]
Number of columns	[5,7]	[6,8]	[7,9]	[8,10]
Relative area of biclusters	6,0%	3,9%	4,8%	4,5%

A second set of datasets was generated to study the efficiency limits of BicSPAM by fixing the number of rows (∣*X*∣=20000) and varying the number of columns (50 ≤∣*Y*∣≤ 200). Background values were generated as the first set of datasets, and 2 biclusters were planted to occupy 5% of the total area.

We rely on match scores (MS) to assess the accuracy of biclustering approaches to recover the planted biclusters. MS(ℬ,ℋ) defines the extent to what found biclusters match with hidden biclusters, while MS(ℋ,ℬ) reflects how well each of the hidden biclusters are recovered.

$$\text{MS}(ℬ,ℋ)=\frac{1}{|ℬ|}\Sigma_{(I_{1},J_{1})\in ℬ}{\text{max}}_{(I_{2},J_{2})\in ℋ}\frac{|I_{1}\cap I_{2}|}{|I_{1}\cup I_{2}|}$$

#### 

*Comparison of biclustering approaches:* four state-of-the-art biclustering approaches were selected: two approaches able to deliver order-preserving biclusters, OPSM [1] and OP-Clustering [7], and two approaches able to discover biclusters under constant, additive and multiplicative models, FABIA with sparse prior Equation [3] and ISA [48]. We used the following software: the BicAT software [49] to run OPSM and ISA approaches and the R package fabia[3]. The default number of iterations for the OPSM method was varied from 10 to 200 iterations. BicSPAM was used with the: *1)* default parameterization, *2)* default parameterization but with sequential patterns gathered from multiple levels of expression (∣*Σ*∣∈{4,7,10}), and *3)* dynamic data-based parameterization. The support threshold for both BicSPAM and OP-Clustering approaches was incrementally decreased 10% and stopped when the output solution had over 50 (maximal) biclusters. We applied FABIA with default parameterizations. The specified number of biclusters for both FABIA and ISA (number of starting points) was the number of hidden biclusters plus 10%: ∣ℋ∣×1.1.

**Figure 10:**
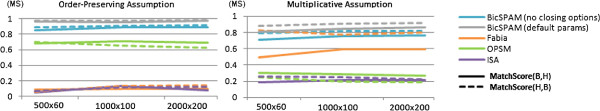
Comparing match scores across biclustering approaches using the generated datasets.

The average performance of these approaches over the synthetic datasets described in Table 1 (with planted biclusters following order-preserving and multiplicative models) is illustrated in Figure 10. OP-Clustering was excluded due to memory problems for the larger datasets. For small datasets, the performance of OP-Clustering is slightly inferior than BicSPAM performance due to the absence of closing and noise-handling options. These results confirm the higher performance of BicSPAM in terms of MS(ℬ,ℋ), that is, the majority of the discovered biclusters are well described by the hidden biclusters (correctness), and MS(ℋ,ℬ), that is, the majority of hidden biclusters can be mapped into a discovered bicluster (completeness). Although OPSM achieves a reasonable performance under the order-preserving assumption, the iterative masking of biclusters degrades the observed match score levels. Additionally, OPSM tends to discover biclusters with varying sizes, which results in a large portion of biclusters with either a very few number of rows or columns. FABIA and ISA approaches are not prepared to discover order-preserving biclusters. However, for the multiplicative coherency, FABIA is a competitive option, although MS(ℬ,ℋ) levels are penalized due to the inclusion of false columns per bicluster. Since order-preserving regularities are more general than multiplicative regularities a penalization in robustness is observed for ISA (prepared to find additive regularities) and OPSM.

#### *Efficiency limits:*

**Figure 11:**
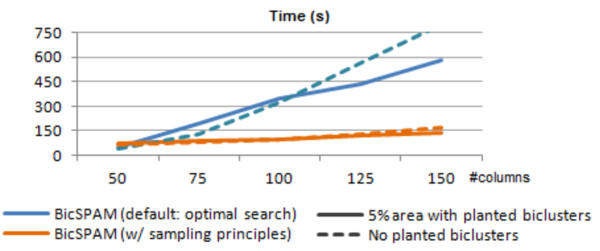
Efficiency of BicSPAM for 20000 rows in the absence and presence of sampling options.

To show the boundaries on BicSPAM efficiency when considering 20.000 rows (magnitude of the human genome), we considered the second set of synthetic data with results provided in Figure 11. BicSPAM support was decreased until a 5% of coverage is achieved. Two scenarios are depicted: one setting where biclusters are planted and another setting without planted biclusters. In the absence of scalability principles, BicSPAM can handle matrices up to 20.000 × 100. In the presence of data sampling principles (according to [50]), BicSPAM can scale for the assessed medium-to-large data settings.

#### *Degree of co-occurrences:*

**Figure 12:**
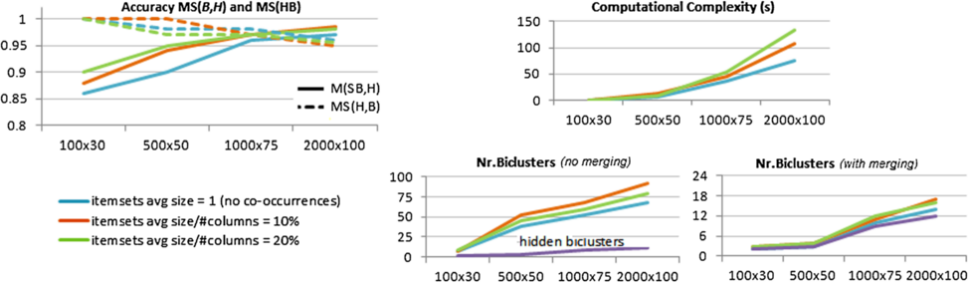
Performance of BicSPAM approaches for datasets with varying properties.

Figure 12 illustrates the performance of BicSPAM over the generated datasets using: the original values (the average number of items per itemset is approximately 1); a discretization to consider an average of 5% of columns per itemset (sequences with 20 itemsets); and a discretization to consider an average of 10% of columns per itemset (sequences with 10 itemsets). These tests were performed using the default parameterizations with no closing options. The retrieved biclusters are shown to match the planted biclusters (MS(ℬ,ℋ) and MS(ℋ,ℬ) above 95% for medium-to-large datasets). These scores are not optimal (100%) due to the exclusion of few rows from the solution as a result of the planted noise or of the allowed overlapping among biclusters. This is also the main reason why the number of discovered biclusters is significantly higher than the number of planted biclusters^c^. As illustrated, this problem is minimized when a merging step (80% overlapping) is considered. Finally, the use of discretization methods decreases the number of precedences, which can lead to a slight decrease in efficiency due to an increase of frequent patterns.

#### *Mining methods:*

**Figure 13:**
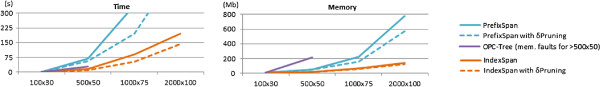
Efficiency of alternative SPM methods across datasets with varying size.

The impact of the algorithmic choice on the efficiency of BicSPAM in terms of time and maximum memory usage is assessed in Figure 13. We used PrefixSpan from SPMF framework [51] and OPC-Tree as the basis of comparison. The impact of mining sequential patterns in the absence and presence of the minimum number of columns per bicluster, *δ* threshold, is presented for a fair comparison. The gains in efficiency from adopting fast database projections are significant, dictating the ability of the SPM task to scale for hard settings. *δ*-based pruning methods also promote efficiency gains. Contrasting with OPC-Tree that requires the full construction of the pattern-tree before the traversal, IndexSpan performs searches with minimal memory waste. For an allocated memory space of 2 GB, we were not able to construct OPC-Trees for input matrices with more than 40 columns.

#### *Pattern representations:*

**Figure 14:**
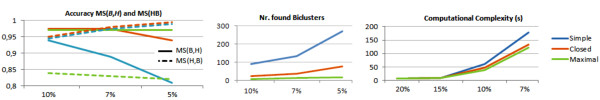
Properties of alternative types of patterns over 1000 × 75 setting for varying levels of support.

The impact of choosing simple, closed and maximal pattern representations is presented Figure 14 for an alphabet length of 10 items and the 1000 × 75 dataset setting. As illustrated, the use of maximal patterns for biclustering should be avoided as it gives preference for biclusters with a large number of columns and discards biclusters with a subset of these columns (even if a larger number of rows is present). This penalizes the MS(ℋ,ℬ) levels. MS(ℬ,ℋ) scores are not so affected as each maximal bicluster is covered by a planted bicluster. Additionally, the use of simple patterns for biclustering can degrade the MS(ℬ,ℋ) in comparison with closed patterns. This score penalizes the discovery of biclusters that are just a part of larger planted biclusters, even when the found biclusters have a heightened homogeneity. The search for closed and maximal patterns slightly increases efficiency. These observations support the use of SPM methods that find closed patterns (corresponding to the notion of maximal biclusters [2]).

#### *Missing values:*

**Figure 15:**
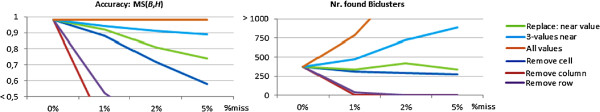
Impact of different techniques to handle missing values for datasets with varying levels of noise.

For the assessment of the proposed strategies to handle *missing values*, we randomly removed a varying number of elements of the generated matrices for the 1000 × 75 setting. Figure 15 illustrates how the performance of BicSPAM (using PrefixSpan with pruning options and 10 items) varies with the percentage of missing elements, which ranges from 0 to 5% (that is, from 0 to 10.000 elements). 5% is already considered a critical number that compromise the ability to retrieve the true biclusters. Three main observations are derived from Figure 15. First, robustness is greater when considering the nearest 2-3 values than when imputing one value only or all the possible values (relaxed strategy). This is due to an increased chance of recovering the original value and, therefore, of not damaging a planted bicluster. When considering all the possible values for a missing element, there is an increased noise added that can lead to the emergence of false biclusters. Second, although removing missing elements (effortless implemented using SPM) is preferred over default options (removal of the columns or of the rows where a missing appears), MS(ℋ,ℬ) score still decreases from 97% to nearly 60% when the percentage of missing values reaches 5%. Third, imputing multiple values penalizes efficiency as the sequence database becomes denser (consistent with the number of found biclusters). Nevertheless, scalability levels are preserved when imputing only the closest 2-3 items for levels of noise up to 5%.

#### *Closing options:*

**Figure 16:**
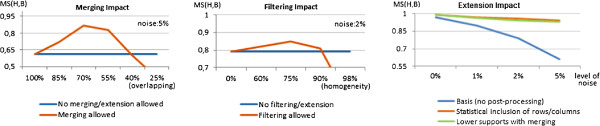
**Impact of *merging * (varying overlapping degrees and 5% of noise), *filtering * (varying homogeneity degrees and 2% of noise) and of *extensions * (varying levels of noise) using the 2000 ×100 setting.**

Varying levels of noise were planted to test the robustness of the proposed closing options. This was performed by replacing the values of specific elements by a new randomly generated value. The percentage of noisy elements were varied from 0 to 10%. We selected the 1000 × 75 setting for this study, the PrefixSpan method, and 20 items for the discretization step. Figure 16 describes the impact of merging, filtering and extension strategies to handle noise.

The impact of *merging* biclusters assuming a 5% level of planted noise is illustrated in Figure 16 (left). The baseline case is when the required overlapping area for merging equals 100% (no merging effect since we are targeting biclusters derived from closed patterns). When relaxing the overlapping criteria, the MS(ℋ,ℬ) levels (and also MS(ℬ,ℋ) levels) increase, as the merging step allows for the recovery of missing columns and rows belonging to planted biclusters. However, this improvement in behavior is only observable until a certain threshold (near 70% for this setting). A correct identification of the optimum threshold can lead to significant gains (near 20 pp for this experimental setting).

The adoption of *filtering* at the row/column level also enhances the ability to recover the planted biclusters. The impact of removing potentially rows and columns (not satisfying an inputed homogeneity threshold) is illustrated in Figure 16 (middle). Filtering is relevant to correct errors related with non-planted co-occurrences when considering restrictive discretizations. Similarly to the merging option, an increase in the matching scores is observed from the baseline case (an homogeneity degree of 0%) up to 75% (given by 1 -*MSR*). From this upper threshold the match scores decrease since the homogeneity criteria becomes too restrictive, which leads to removal of rows and columns from planted biclusters due to a misinterpretation of their natural levels of noise.

Finally, the impact of different *extension* strategies is illustrated in Figure 16 (right). When increasing the planted noise, the presence of the extension options it is critical to maintain attractive levels of accuracy. Both the inclusion of new rows and columns recurring to statistical analyzes or by lowering the support of SPM methods and merging the resulting biclusters are able to maintain match score levels above 90% (30 pp higher than the baseline case).

#### *Symmetries:*

**Figure 17:**
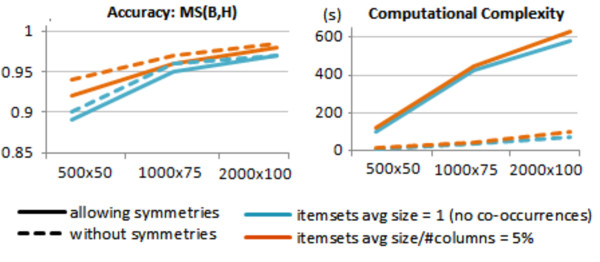
Difficulty of mining order-preserving biclusters with and without symmetries.

Figure 17 describes how mining symmetric behavior with BicSPAM compares with the default BicSPAM behavior (dashed lines). For this evaluation, we varied the sign of some rows for each planted bicluster. The default BicSPAM (no symmetries) was tested over the same matrices but using planted biclusters without symmetries. MS(ℬ,ℋ) levels are preserved. The observed differences in accuracy are related with the higher probability of background values to form a non-planted order-preserving bicluster when considering symmetric behavior (validated by the high number of found biclusters). Finally, the impact of using symmetries in the time complexity is considerably less than the expected ∣ *Y* ∣ times due to the implemented heuristics to prune the number of iterations.

### Results in real data

To assess the relevance of BicSPAM results over biomedical contexts, we selected four distinct datasets: dlbcl (180 columns/conditions, 660 rows/genes) [52], yeast (18 columns, 2884 rows) [53], colon cancer (62 columns, 2000 rows) [54] and leukemia (38 columns, 7129 rows) [55]. These datasets have been previously used by biclustering approaches with flexible coherency criteria [1,3,13].

**Figure 18:**
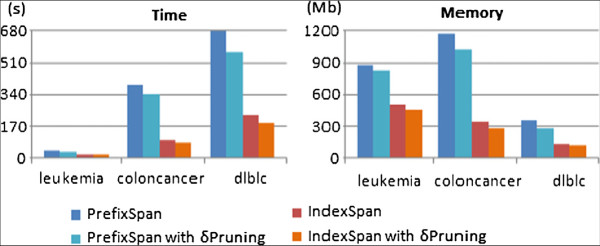
Efficiency of BicSPAM over real data.

**Figure 19:**
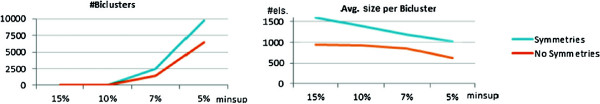
Flexibility of order-preserving solutions with symmetries for varying dataset settings.

Figure 18 compares the performance of the extended IndexSpan method when considering a discretization alphabet of 20 items, *θ* = 8% and *δ *= 5. This analysis reinforces the derived observations from synthetic data.Figure 19 illustrates the impact of including symmetries when mining the yeast dataset. We applied BicSPAM with an overall normalization followed by a Gaussian discretization with 20 items. The shown solutions rely on closed patterns and exclude identical biclusters. Interestingly, we can see that order-preserving solutions that allow for symmetric behavior are able to capture a higher number of biclusters with larger sizes on average. This is an indicator of superior flexibility, which is related with the integrated capturing of regulatory and co-regulatory behavior.

#### 

*Biological relevance:* To assess the *biological relevance* of the discovered order-preserving biclusters, the statistical relevance was obtained using Gene Ontology (GO) annotations recurring to the GoToolBox [56]. To perform the analysis for functional enrichment we computed the *p*-values using the hypergeometric distribution to access the over-representation of a specific GO term. In order to consider a bicluster to be highly significant, we require its genes to show significant enrichment in one or more of the “biological process” ontology terms by having a Bonferroni corrected *p*-value below 0.01. A bicluster is considered significant if at least one of the GO terms is significantly enriched by having a *p*-value below 0.05.

**Figure 20:**
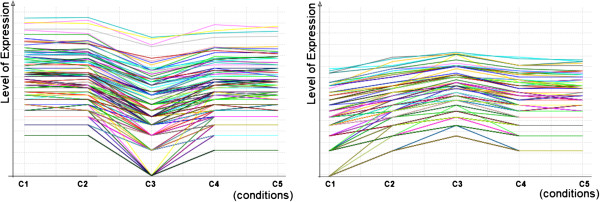
Two order-preserving biclusters with small number of conditions for the yeast dataset.

We were able to derive an average of 68 significant (and non-similar) biclusters using BicSPAM with default parameterizations across datasets when considering a minimum number of *δ *= 5 conditions. Two illustrative order-preserving biclusters discovered in the yeast dataset are shown in Figure 20.

In particular, the average number of significant biclusters increases to over 80 biclusters with a larger number of elements in average when considering symmetries. This is a critical observation since it means that there are groups of genes with biological relevance that can only be discovered through biclustering under a flexible order-preserving setting when symmetries are considered.

Table 2 provides an illustrative set of the found order-preserving biclusters with statistical significance. The properties of the biclusters with biological significance are dependent on the type of dataset, number of items (with impact on the number of precedences) and on the allowed closing options.

**Table 2:** Illustrative biclusters passing the GO term-enrichment test at 1% and 5% significance levels after Bonferroni correction

**Dataset**	***♯*** **Genes**	***♯*** **Conds**	***♯*** **Preced.**	***♯*** **Items**	**Notes**	***♯*** **p-values**	***♯*** **p-values**	**Best**
						**<0 *. *** **01**	**[0.01,0.05]**	**p-value**
Dlbcl	179	6	4	20	No closing options	5	2	3.12E-4
Dlbcl	207	9	5	25	Merging allowed	6	1	2.33E-5
Yeast	167	5	3	10	No closing options	11	3	2.12E-4
Yeast	240	8	4	15	Extensions allowed	10	1	7.13E-7
Colon	769	6	4	25	Merging allowed	12	2	6.08E-8
Leukemia	1645	6	3	20	Extensions allowed	9	2	3.47E-9

## Conclusions

Pattern-based approaches for order-preserving biclustering are proposed with the goal of performing efficient exhaustive searches under flexible conditions. Results support their ability to find highly flexible and robust solutions over matrices with sizes up to 20000 rows and 200 columns. Results in both synthetic and real data show that BicSPAM can surpass the drawbacks identified for existing order-preserving approaches, namely more relaxed scalability boundaries, flexible expression profiles, and superior robustness to noise and missing values.

BicSPAM makes available dynamically parameterizable options dependent on the input data context. BicSPAM allows: 

• different SPM methods, pattern representations (as simple, condensed and approximate), and dynamic optimizations to seize the specificities of the input datasets;

• multiple options to deal with noise and missing values according to different relaxation levels;

• arbitrary number of items and different discretization options (including strategies to deal with the items-boundary problem) with heightened influence on the solution;

• multiple ways to deal with the composition of flexible structures and with the numerosity of biclusters through extension-merging-filtering steps without the need to adapt the core task.

Furthermore, this work introduces the notion of order-preserving biclusters with symmetries and proposes an efficient method for their effective discovery. Results reveal that allowing symmetries is critical to simultaneously capture activation and regulatory mechanisms within a biological process.

As future work, we expect to adapt the mining step to search for lengthy sequential patterns by merging smaller sequential patterns discovered under greater support thresholds according to colossal pattern mining principles [47]. This direction also promotes the scalability of BicSPAM. Finally, we expect to integrate contributions from constraint-based pattern mining in BicSPAM to support knowledge-guided biclustering in biological contexts.

## Software availability

The used datasets and BicSPAM executables are available in http://web.ist.utl.pt/rmch/software/bicspam.

## Endnotes

^a^ Greedy iterative searches rely on the selection, addition and removal of rows and columns until the merit function is maximized locally [1,57,58]. Exhaustive searches use merit functions to guide the space exploration [18,59]. Approaches that combine clusters from both dimensions use similarity metrics (the merit functions) for the clustering and joining stages [60,61]. Divide-and-conquer searches exploit the matrix recursively using a global merit function [62]. Stochastic approaches assume that biclusters follow multivariate distributions [3,8,63] and learn their parameters by maximizing a likelihood (merit) function.

^b^ Existing order-preserving search paradigms also vary with regards to the number of output biclusters – either parameterized (existing greedy approaches) or undefined (existing exhaustive approaches) – and to the number of search iterations – either one bicluster at a time (existing greedy approaches) or all biclusters at a time (existing exhaustive approaches).

^c^MS(ℋ,ℬ) reveals how the hidden biclusters were covered by the nearest found biclusters. Since there is at least one found bicluster with a direct correspondence to each hidden bicluster, BicSPAM has MS(ℋ,ℬ) levels generally higher than MS(ℬ,ℋ).

## Competing interests

The authors declare that they have no competing interests.

## Authors’ contributions

All the authors were involved in the design of the solution and in the writing of the manuscript. All authors read and approved the final manuscript.
